# Comparative Evaluation of Six Traditional Fermented Soybean Products in East Asia: A Metabolomics Approach

**DOI:** 10.3390/metabo9090183

**Published:** 2019-09-13

**Authors:** Yong Sung Kwon, Sunmin Lee, Seung Hwa Lee, Hae Jin Kim, Choong Hwan Lee

**Affiliations:** 1 Department of Bioscience and Biotechnology, Konkuk University, Seoul 05029, Korea; michaelsan@hanmail.net (Y.S.K.); duly123@naver.com (S.L.); 2 Experiment Research Institute, National Agricultural Products Quality Management Service, Gyeongsangbuk-do 39660, Korea; shlee96@korea.kr (S.H.L.); asarela00@korea.kr (H.J.K.)

**Keywords:** fermented soybean product, metabolomics, antioxidant activity

## Abstract

Many ethnic fermented soybean products (FSPs) have long been consumed as seasoning and protein sources in East Asia. To evaluate the quality of various FSPs in East Asia, non-targeted metabolite profiling with multivariate analysis of six traditional FSPs (Natto; NT, Cheonggukjang; CG, Doenjang; DJ, Miso; MS, Doubanjiang; DB, Tianmianjiang; TM) was performed. Six FSPs could be clearly distinguished by principle component analysis (PCA) and partial least square-discriminant analysis (PLS-DA). Amino acid contents were relatively higher in NT and CG, sugar and sugar alcohol contents were relatively higher in MS and TM, isoflavone glycoside contents were relatively highest in CG, isoflavone aglycon contents were the highest in DJ, and soyasaponin contents were the highest in CG. Antioxidant activity and physicochemical properties were determined to examine the relationships between the FSPs and their antioxidant activities. We observed a negative correlation between isoflavone aglycon contents and 2,2’-azino-bis(3-ethylbenzothiazoline-6-sulphonic acid) (ABTS) activity. Furthermore, the order of ABTS activity of FSPs has a positive correlation with the order of soybean content in the six FSPs. Herein it was found that primary metabolites were affected by the main ingredients and secondary metabolites were most influenced by the fermentation time, and that soybean content contributed more to antioxidant activity than fermentation time.

## 1. Introduction

Fermented food has attracted considerable attention due to its potential health benefits. Fermentation has long been utilized to preserve and enhance the storage life, texture, flavor, digestion efficiency, and functional properties of food [1].

Soybean, first grown in East Asia thousands of years ago [2], is an important crop with abundant nutrients; however, the nutritive level of soybean could be restricted by the presence of several anti-nutritional and toxic substances such as trypsin inhibitor and phytate [3]. Fermentation approaches have overcome these limitations and enhanced the quality of soybean in various ways, such as increasing digestibility and enhancing nutritive levels [4]. Many ethnic fermented soybean products (FSPs) have long been consumed as a seasoning and protein source in East Asia. For example, Korean FSPs are *cheonggukjang*, *doenjang*, and *gochujang*; Japanese FSPs are *natto* and *miso*, while Chinese FSPs are *doubanjiang*, *tianmianjiang*, *douche*, and soy sauce [5]. These FSPs are affected by numerous factors, such as the type of raw materials, microorganism used in fermentation, fermentation time, and fermentation temperature [6,7]. The health benefits of these FSPs include anti-breast cancer effects [8], anti-inflammatory activity [9], anti-oxidant activity [10], anti-mutagenic effect [11], anti-diabetic effect [12], and anti-neuroinflammatory effect [13]. Many researchers have reported that isoflavone and peptides are the bioactive compounds present in FSPs that are responsible for these health benefits [9,14].

Recent studies on FSPs have elucidated the effects of manufacturing process [15] and fermentation time [16] on the change in total metabolite content. Further, the study on multiple FSPs focused only on the content of isoflavone, a well-known bioactive compound in FSPs [10]. The untargeted metabolite profiles and their functional aspects are largely unknown and elusive for many FSPs. Notably, the primary metabolites contribute to the food savor and nutrition, while the secondary metabolites influence the associated bioactivities. Hence, we argue the comprehensive analyses of both primary and secondary metabolomes in most revered FSPs available in the market. The untargeted metabolomic studies involving commercial FSP’s may unravel the biochemical aspects of associated nutritional and functional activities and may further act as biomarkers for their quality standardization. Metabolomics is a useful tool for analyzing metabolite changes during fermentation of fermented food such as *black tea*, *green tea*, *kimchi*, and *crab paste* [17,18,19,20]. Thus, metabolomic technologies generate large complex datasets and therefore need advanced statistical and bioinformatic tools to assist in data interpretation [21]. Non-targeted metabolomics analysis may be useful for comparing and analyzing general metabolites in various FSPs.

Herein, the objectives of this study are to evaluate the quality of traditional FSPs (*natto*, *cheonggukjang*, *doenjang*, *miso*, *doubanjiang*, and *tianmianjiang*) via metabolomics approach and antioxidant activity, and to elucidate the relationships between the metabolites and the antioxidant activity of FSPs in East Asia. We performed non-targeted metabolite profiling using multivariate analysis of six FSPs. 

## 2. Results and Discussion

### 2.1. Metabolomic Profiling Combined Multivariate Analysis

Soybeans were cultured directly with *Aspergillus* spp., *Rhizopus* spp., or *Mucor* spp. or *Bacillus subtilis* to yield more digestive and/or tasty foods in FSPs. Furthermore, cooked soybeans were mixed with some cereal grains cultured with *Aspergillus* spp. [22]. Many researchers have reported that NT, CG, DJ, MS, DB, and TM included *Aspergillus oryzae* or *Bacillus subtilis* [23,24,25]. Typically, substrates of FSPs were hydrolyzed by extracellular enzymes like protease, α-amylase, and lipase, obtained from microorganism such as *B. subtilis* and *A. oryzae* during fermentation [26,27].

To investigate metabolite states, metabolite profiling of the thirty FSPs (NT, CG, DJ, MS, DB, and TM) was performed using gas chromatography time-of-flight mass spectrometry (GC-TOF-MS) and ultrahigh-performance liquid chromatography linear trap quadrupole ion trap tandem mass spectrometry (UHPLC-LTQ-IT-MS/MS) combined multivariate analysis. 

#### 2.1.1. Primary Metabolites of Six FSPs

**Figure 1:**
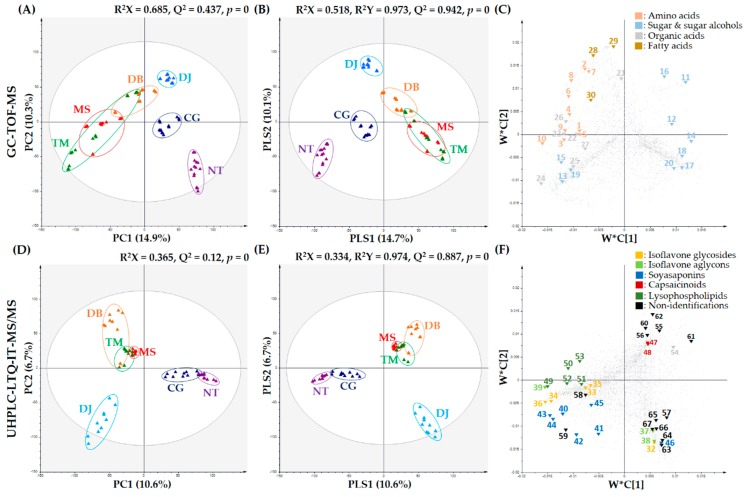
PCA score plots, PLS-DA score plots, and PLS loading plots of six FSPs (NT: *natto*, CG: *cheonggukjang*, DJ: *doenjang*, MS: *miso*, DB: *doubanjiang*, TM: *tianmianjiang*). (**A**) PCA score plot, (**B**) PLS-DA score plot, and (**C**) PLS-DA loading plot derived from GC-TOF-MS; and (**D**) PCA score plot, (**E**) PLS-DA score plot, and (**F**) PLS-DA loading plot derived from UHPLC-LTQ-IT-MS/MS. Plot annotation: 1, serine: 2, threonine: 3, alanine: 4, glycine: 5, isoleucine: 6, phenylalanine: 7, leucine: 8, methionine: 9, valine: 10, proline: 11, ribose: 12, ribitol: 13, maltose: 14, xylitol: 15, glycerol: 16, galactose: 17, myo-inositol: 18, sucrose: 19, N-acetyl-D-glucosamine: 20, pinitol: 21, glutaric acid: 22, gluconic acid: 23, fumaric acid: 24, acetic acid: 25, benzoic acid: 26, ribonic acid: 27, lactic acid: 28, linolenic acid: 29, stearic acid: 30, glyceryl palmitate: 31, hydroxylamine: 32, genistein derivatives: 33, daidzein derivatives: 34, genistin: 35, malonyldaidzin: 36, malonylgenistein: 37, glycitein: 38, genistein: 39, daidzin: 40, dehydrosoyasaponin I: 41, soyasaponin III: 42, soyasaponin IV: 43, soyasaponin V: 44, soyasaponin I: 45, soyasaponin II: 46, soyasapogenol C: 47, capsaicin: 48, dihydrocapsaicin: 49, lysoPE16:0: 50, lysoPE18:1: 51, lysoPC18:2: 52, lysoPC18:3: 53, lysoPC18:1: 54, flazin: N.I., non-identified compound: 55, N.I. 1: 56, N.I. 2: 57, N.I. 3: 58, N.I. 4: 59, N.I. 5: 60, N.I. 6: 61, N.I. 7: 62, N.I. 8: 63, N.I. 9: 64, N.I. 10: 65, N.I. 11: 66, N.I. 12: 67, N.I. 13.

To investigate metabolite state, metabolite profiling of the six FSPs (NT, CG, DJ, MS, DB, and TM) was performed using GC-TOF-MS combined multivariate analysis. The PCA score plot derived from GC-TOF-MS analysis indicated that thirty samples were clustered according to FSP type (Figure 1A). MS, DB, and TM were divided into DJ, CG, and NT, based on PC1 (14.9%). TM was clustered with MS and DB, whereas DJ, CG, and NT were clustered independently. Therefore, it can be concluded that NT, CG, and DJ were composed of only soybean as the general main ingredient and MS, DB, and TM contained cereal (rice or wheat) as the general main ingredients (Table 1). 

The PLS-DA score plot derived from GC-TOF-MS analysis showed a pattern similar to the one obtained by the PCA analysis (Figure 1B). Metabolite patterns by main ingredients in six FSPs were tentatively identified based on their variable importance in projection values (VIP > 1.0) and *p*-value (*p* < 0.05). Thirty-one metabolites, including 10 amino acids, 10 sugar and sugar alcohols, 7 organic acids, 3 fatty acids, and hydroxylamine were determined to be important variables by PLS1 (14.7%) and PLS2 (10.1%) (Table S2).

**Figure 2:**
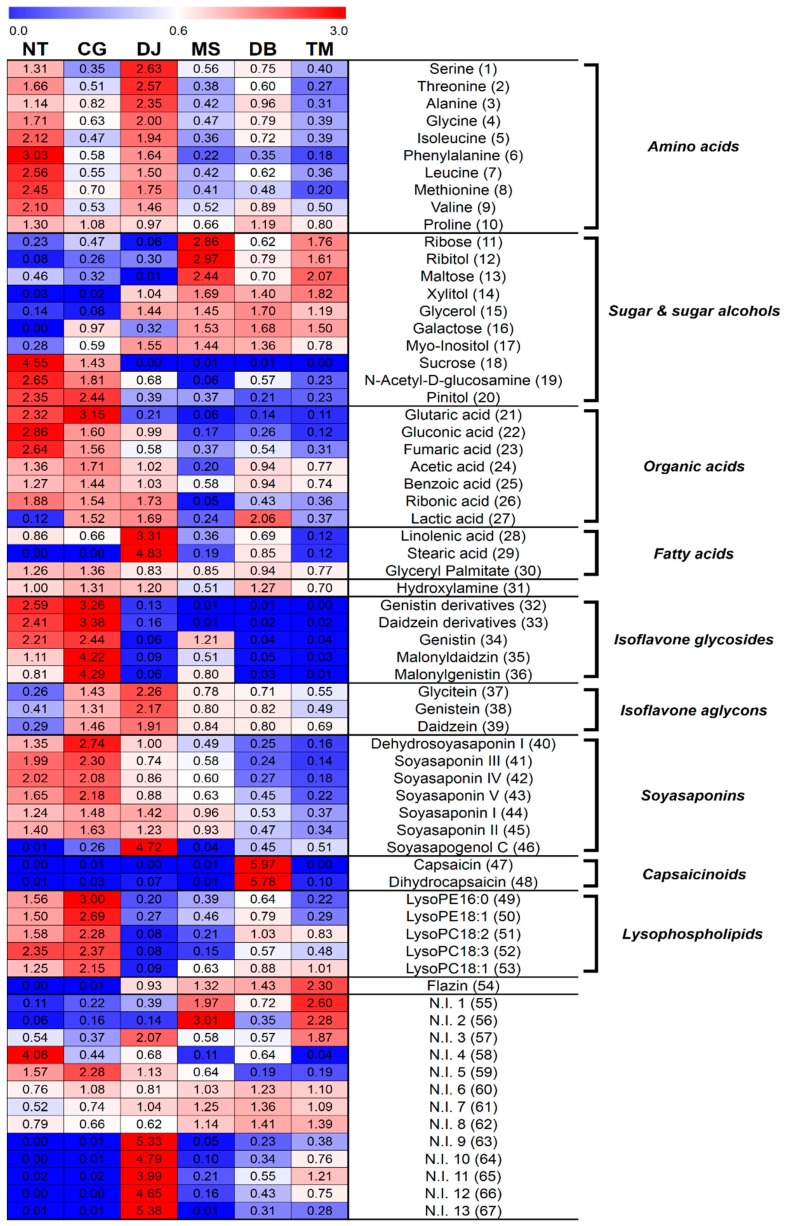
Heat map representations for the relative contents of significantly discriminant primary and secondary metabolites in six FSPs analyzed by GC-TOF-MS and UHPLC-LTQ-IT-MS/MS. Metabolites were selected by variable importance in the projection (VIP) value > 1.0, *p*-value < 0.05. All values were averaged for FSP types.

The relative contents of the metabolites are shown in Figure 2. The heat map color (blue-to-red) represents the relative fold-change-normalized values for each metabolite content, and all the values were averaged for FSP types.

The relative contents of amino acids (1–10) were higher in NT and DJ than other FSPs. The amino-type nitrogen contents were highest in DJ, followed by NT. In contrast, titratable acidity was lowest in NT and CG, and pH was highest in NT, followed by CG (Table 2). Herein, we conjecture that NT and DJ, which contain relatively higher soybean content, have relatively higher amino acid contents, because the relatively higher protein content of soybean was degraded by protease from microorganisms during fermentation. Amino acids also contribute to the taste of foods. Serine, threonine, alanine, glycine, and proline are related to the sweet taste, while isoleucine, phenylalanine, leucine, methionine, and valine contributed to the bitter taste [28]. Organic acids (23–26) were observed mainly in NT, CG, and DJ. The generation of organic acids rely on the inoculation of the microbial strains utilized in the preparation of FSPs, and the organic acids contribute to the taste and aroma in FSPs.

Conversely, sugar and sugar alcohols (11–17) were primarily associated with MS, DB, and TM. Concordantly, the reducing sugar contents were also highest in MS and TM (Table 2). On the other hand, we inferred that MS, DB, and TM, containing relatively higher cereal (wheat or rice) contents, have relatively higher sugar and sugar alcohol contents because the relatively higher carbohydrate content of cereal (wheat or rice) was degraded by α-amylase from microorganisms during fermentation. In addition, these sugar and sugar alcohols reportedly enhanced the mouth-feel and sweet flavor [29]. Relative contents of fatty acids (28, 29) were highest in DJ. 

We inferred that the primary metabolites in the six FSPs may be largely affected by the general main ingredients of FSP, due to biotransformation of proteins and carbohydrates in the main ingredients to small molecules like amino acids and sugar.

#### 2.1.2. Secondary Metabolites of Six FSPs 

Metabolite profiling of six FSPs (NT, CG, DJ, MS, DB, and TM) was performed using UHPLC-LTQ-IT-MS/MS combined multivariate analysis. The PCA score plot indicated that thirty samples were clustered according to FSP type. NT and CG were divided into MS, DB, TM, and DJ based on PC1 (10.6%) and PC2 (6.7%) (Figure 1D). MS and TM were clustered close to each other, but DB was clustered independently, while CG and NT were clustered close to each other. The fermentation period of NT and CG was under 3 days, the fermentation period of MS, DB, and TM was approximately 1 year, and that of DJ was 1-3 years (Table 1). This pattern based on PC2 was similar to that based on PC1 of the PCA derived from GC-TOF-MS analysis (Figure 1A). 

The PLS-DA score plot derived from UHPLC-LTQ-IT-MS/MS analysis (Figure 1E) showed a pattern comparable to the one obtained from PCA analysis. Thirty-six metabolites, including 8 isoflavonoids, 7 soyasaponins, 2 capsaicinoids, 5 lysophospholipids, flazin, and 13 non-identified metabolites were selected based on variable importance in projection values (VIP > 1.0) and *p*-value (*p* < 0.05) (Table S2). 

The corresponding loading plots (Figure 1F) and heat map (Figure 2) showed that the contents of isoflavone glycosides (32–36) were higher in NT and CG than other FSPs, whereas relative isoflavone aglycon (37–39) content was the highest in DJ. Soybean is reportedly one of the plants containing the highest isoflavone contents [30]. In soybean, isoflavones were observed mainly as glycosides or their respective malonates or acetyl conjugates [10]. These isoflavones were hydrolyzed to obtain isoflavone aglycons by enzymes like β-glucosidase and esterase secreted from microorganisms like *Bacillus* spp., *Aspergillus* spp., etc. during fermentation [31,32]. In many studies, the amount of isoflavone glycosides tended to decrease and that of isoflavone aglycons tended to increase during fermentation to obtain FSPs [6,16,33].

In this study, relative contents of soyasaponins (40–45) of six FSPs generally followed the order CG > NT > DJ > MS > DB > TM (Figure 2), similar to the order of soybean content (NT > CG > DJ > MS > DB > TM) (Table 1), except that soyasapogenol C (46) content was highest in DJ (Figure 1F, Figure 2) because of the longest fermentation period of DJ. Soyasapogenols were hydrolyzed by saponin hydrolase from *Aspergillus oryzae* [34]. According to Kamo et al., soyasapogenol content was higher in FSPs with relatively long fermentation periods [35]. Further, according to Gurfinkel et al., soyasapogenols (aglycon) are normally more bioactive than the corresponding glycosides [36]. The soyasaponin aglycons are less astringent than soyasaponins [37].

The fermentation periods of NT (18 h) and CG (2 days) were relatively shorted than that of DJ (1-3 years) (Table 1). Thus, metabolites in NT and CG could be a slightly biotransformed by organism. In contrast, metabolites in DJ, having the longest fermentation period, may be considerably biotransformed by microorganisms. Hence, we concluded that biotransformation of isoflavone and soyasaponin derivatives may be affected by the fermentation period.

Some saponins including soyasaponin and sapogenol reportedly contribute to the bitter or astringent taste [37], and have various biological effects, including lowering cholesterol [38], anti-cancer effect [39] and anti-inflammatory effect [40]. 

Relative flazin (54) content was the highest in TM and flazin was not detected in NT and CG (Figure 2). It reportedly inhibited the proliferation of human promyelocytic leukemia cells [41], and according to Yun-Hua Wang et al., shows weak anti-HIV-1 activity [42]. Flazin was previously identified in fermented foods like soy sauce, sake, and tamari [41,43], and was reportedly a metabolic product of *Pediococcus halophilus*, which is essential for soy sauce fermentation [44]. Numerous studies reported that *Bacillus* species was the overwhelmingly dominant type of microorganism in NT and CG [23,45,46]. CG and NT are inoculated by *Bacillus* species, and do not participate in fermentation after adding salt (but NT does not have a salt treatment process) [47,48]. On the other hand, manufacturing processes for DJ, MS, DB, and TM were more complex than those for NT and CG. The manufacturing process in DJ, MS, DB, and TM included solid state fermentation such as *koji* and *meju*. After salt treatment processes, such as brining and adding salt, they are continuously fermented [22,48,49,50]. Therefore, their microbial community is more complex than that of NT and CG. Hence, we assumed that the difference in relative flazin content in six FSPs was influenced by the disparity in microbial community among the six FSPs.

The heat map showed that capsaicinoids (47, 48) were only detected in DB (Figure 2) because chilli pepper is the general ingredient of DB. Capsaicinoids are mainly found in the *Capsicum* plant [51] and typically contribute to the hot pungent taste [52]. 

Relative contents of lysophospholipids (49–53) were high in NT and CG (Figure 1F and Figure 2). 

We compared the secondary metabolite states in the six FSPs. The relative contents of isoflavone glycosides and soyasaponins were highest in CG, whereas the contents of isoflavone aglycons and soyasapogenol C were highest in DJ. The relative contents of capsaicinoids and flazin were highest in DB and TM, respectively. Accordingly, the secondary metabolite state in six FSPs may be affected by various factors, such as main ingredients, fermentation period, and microbial community.

### 2.2. Correlation Assay between Bioactive Phenotypes and Metabolites

**Figure 3:**
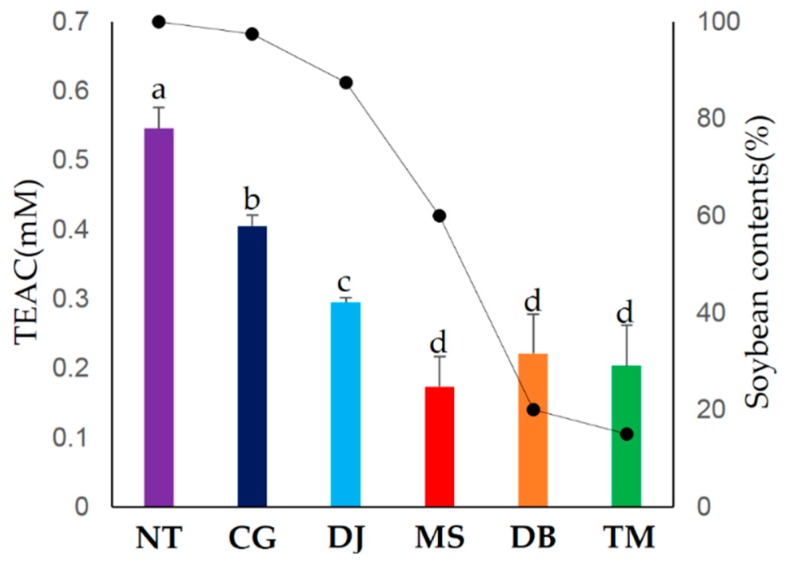
Comparison of ABTS (bar graph) and soybean content (line graph) for six FSPs. All the values were averaged for the six FSP types. Different letters in the table indicate the significant difference obtained by ANOVA followed by Duncan’s multiple-range test (*p*-value < 0.05).

To compare the antioxidant activities of six FSPs, we measured ABTS (Figure 3), total flavonoid content (TFC), and total phenolic content (TPC) (Table 2). The ABTS value was the highest in NT, followed by CG > DJ > MS = DB = TM (Figure 3). The TFC and TPC values were highest in NT, whereas there was less significant difference among the six FSPs (Table 2). 

To evaluate the contribution of metabolites to soybean content, antioxidant activity, and physicochemical properties, we conducted correlation analysis. Interestingly, we observed a negative correlation between isoflavone aglycons (37–39) and ABTS and a positive correlation between isoflavone glycosides (32–36) and ABTS (Figure S1).

Typically, isoflavone is renowned as a representative antioxidant in soybean. Multiple hydroxyl groups in the flavonoid structure can inhibit oxidation. The flavonoid aglycons have stronger antioxidant activity than their respective glucosides. Many researchers have reported that isoflavone glycosides were hydrolyzed to their aglycons and the antioxidant activity of FSPs tends to increase with increasing fermentation time [16,53].

According to Lee et al., the antioxidant activity difference between aglycons and glycosides in isoflavone is significantly less than those of well-defined antioxidants, epicatechins and α-tocopherol [54]. Moreover, Lee et al. reported that although flavonoids have much higher antioxidant capacities than amino acids, the latter contributed more to the antioxidant capacity of *gochujang* than flavonoids due to the large amount of amino acids [55]. This study also revealed a positive correlation between amino acids and ABTS (Figure S1). Although the main antioxidant in soybean is isoflavone aglycons, these metabolites could not affect the antioxidant activity of the six FSPs. Since the order of ABTS activities is similar to that of soybean content (Figure 3), soybeans may contain antioxidants including isoflavone derivatives and amino acids in the six FSPs. Thus, we assumed that the antioxidant activity of FSPs is related more to soybean content than to fermentation period.

## 3. Materials and Methods

### 3.1. Chemicals and Reagents

HPLC-grade water, methanol, and acetonitrile (HPLC grade and Optima LC/MS grade) were purchased form Fisher Scientific (Pittsburgh, PA, USA). Formic acid, methoxyamine hydrochloride, pyridine, N-methyl-N-(trimethylsilyl)trifluoroacetamide (MSTFA), potassium persulfate, 2,21-azinobis(3-ethylbenzothiazoline-6-sulfonic acid) diammonium salt (ABTS), Folin–Ciocalteu’s phenol, sodium hydroxide, acetic acid, formaldehyde solution 37%, 6-hydroxy-2,5,7,8-tetramethylchroman-2-carboxylic acid (Trolox), gallic acid, and naringin were obtained from Sigma-Aldrich (St. Louis, MO, USA). Sodium carbonate and diethylene glycol were obtained from Junsei Chemical Co., Ltd. (Tokyo, Japan). 2-Chlorophenylalanine was obtained from Santa Cruz Biotechnology, Inc. (Dallas, TX, USA).

### 3.2. Sample Preparation

#### 3.2.1. Sample Information

Total thirty traditional FSPs, with five of each kind, including 10 Korean FSPs (5 *doenjang* and 5 *cheonggukjang*), 10 Chinese FSPs (5 *doubanjiang* and 5 *tianmianjiang*), and Japanese FSPs (5 *natto* and 5 *miso*) were purchased from the market. All the samples, except NTs, were stored at 4 °C and the NTs were stored under freezing conditions (−24 °C) until analysis. Sample information is shown in Table 1, Table S3. Additionally, the content range of ‘General main ingredient’ was mainly based on the ingredient labels of FSPs. Some product didn’t have the ingredient label. In this case, we referred to several textbook and other studies. The ranges of the ‘General fermentation period’ were also obtained from several textbooks and other studies. [22,33,47,50,56,57].

#### 3.2.2. Sample Extraction and Derivatization for Metabolite Profiling

All the FSP samples were lyophilized for 3 days and powdered, and extracted with 80% aqueous methanol (100 mg/mL) at room temperature in a 2-mL safe-lock tube using a Mixer Mill (Retsch GmbH & Co, Germany) at 30 Hz/s for 150 s. The sample mixtures were then centrifuged at 8000× *g* for 8 min at 4 °C and the supernatants were filtered using a 0.2-µm polytetrafluoroethylene (PTFE) filter. Next, the samples were dried in a speed vacuum concentrator (Biotron, Seoul, Korea). The dried samples were dissolved in 80% methanol, containing 2-chlorophenylalanine as the internal standard, to a final concentration of 40,000 ppm (100 mg/mL) and filtered. Then, the resolved sample analytes were diluted up to 10,000 ppm (10 mg/mL). For the GC-TOF-MS analysis, the resolved sample analytes were completely dried using a speed vacuum concentrator and derivatized. Derivatization occurred over two steps. The first step was oximation, conducted by adding the dried sample with 50 µL of methoxyamine hydrochloride in pyridine (20 mg/mL) and incubating the reaction at 30 °C for 90 min. The second step was silylation, performed by supplementing with 50 µL of MSTFA and incubating the reaction at 37 °C for 30 min, followed by filtration.

### 3.3. GC-TOF-MS Analysis

The gas chromatography time-of-flight mass spectrometry analysis was performed on an Agilent 7890A gas chromatograph system (Santa Clara, CA, USA) with a Pegasus HT TOF-MS (Leco Corp., St. Joseph, MI, USA). A Rtx-5MS (30 m length, 0.25 mm inner diameter, J&W Scientific, Folsom, CA, USA) was used to provide the carrier gas helium (He) at a constant flow rate of 1.5 mL/min. Each derivatized sample (1 µL) was injected into the GC-system under split mode (5:1). The injector and ion source temperatures were keeped at 250 °C and 230 °C, respectively. The oven temperature was set at 75 °C for 2 min and then increased to 300 °C at 15 °C/min, which was sustained for 3 min. The detector voltage was 1650 V, and mass scan range was 50–1000 m/z. Three analytical replications were conducted for each FSP sample.

### 3.4. LC-MS Analysis

Ultra-high-performance liquid chromatography linear trap quadrupole ion trap tandem mass spectrometry analysis for all FSP sample analyte was conducted using a Thermo Fisher Scientific LTQ ion trap mass spectrometer equipped with an electrospray interface (Thermo Fisher Scientific, San Jose, CA, USA) and Dionex UltiMate 3000 RS autosampler, RS column compartment, RS diode array detector, and RS pump (Dionex Corporation, Sunnyvale, CA, USA). A Thermo Scientific Syncronis C18 UHPLC column (100 mm × 2.1 mm i.d.: 1.7 μm particle size) was used to separate a 10-μL sample analyte. The mobile phase consisted of water (solvent A: 0.1% formic acid, *v*/*v*) and acetonitrile (solvent B: 0.1% formic acid, *v*/*v*) pumped through binary solvent delivery system at a flow rate of 0.3 mL/min. The solvent gradient program was as follows: 10% B for 1 min, increased to 100% B for 14 min, maintained for 3 min, and decreased to 10% B in 1 min, and maintained at 10% B for the final 3 min. Acquisition range for photodiode array and detection range for mass spectrometry in both positive and negative ion mode was 200–600 nm wavelength and 100–1000 m/z for all FSP sample extracts. The MS operating parameters were as follows: capillary temperature, 275 °C; source voltage, ±5 kV; and capillary voltage, 39 V. 

A robust mass analyzer, Orbitrap, was used to acquire a high-resolution mass spectrum and elemental composition. The analysis was performed on an UHPLC system on a vanquish binary pump (Thermo Fisher Scientific, Waltham, MA, USA) equipped with auto-sampler and column compartment. Chromatographic separation was conducted on Phenomenex KINETEX^®^ C18 column (100 mm × 2.1 mm, 1.7 µm particle size; Torrance, CA, USA) and the injection volume was 5 µL. The column temperature was maintained at 40 °C and the flow rate was 0.3 mL/min. The mobile phase consisted of 0.1% formic acid in water (Solvent A) and 0.1% formic acid in acetonitrile (Solvent B). The gradient parameters were set as follows: 5% solvent B was flown initially for 1 min, followed by a linear increase to 100% solvent B over 9 min, sustained at 100% solvent B for 1 min, followed by a gradual decrease to 5% solvent B over 3 min. The total running time was 14 min. The MS data were collected in the range *m*/*z* 100–1000 (in negative and positive ion mode) using an Orbitrap Velos Pro^TM^ system, which is combined with an ion trap mass spectrometer (Thermo Fisher Scientific, Waltham, MA, USA) coupled with and HESI-II probe. The probe heater and capillary temperatures were set at 300 °C and 350 °C, separately. The capillary voltage was set at 3.7 and 2.5 kV in the positive and negative ion modes. 

### 3.5. Data Processing and Multivariate Statistical Analysis

The raw data (GC−TOF−MS, UHPLC−LTQ−IT−MS/MS) were converted to netCDF (*.cdf) format using LECO Chroma TOF (Version 4.4, LECO Corp., St. Joseph, MI, USA) and Thermo Xcalibur software (version 2.1, Thermo Fisher Scientific, Waltham, MA, USA). The respective netCDF (*.cdf) files were aligned the MetAlign software (http://www.metalign.nl) for data processing, as reported by Won et al. [58]. The resulting data matrix, which included the corrected peak mass (*m*/*z*), retention times (min), and peak area information as variables, was determined using SIMCA-P+ software (version 15.0.2, Umetrics, Umea, Sweden) for multivariate statistical analysis by principal component analysis (PCA), partial least squares discriminant analysis (PLS-DA). Also, PLS-DA loading plot interpretation were conducted to the metabolic disparities between FSP. The putative metabolite identification was performed by matching molecular weights, formula, retention time, mass fragmentations, and UV absorbance data available in the published references, the chemical dictionary version 7.2 (Chapman and Hall/CRC), and in-house library (off-line database in laboratory made by analyzing standards) (Tables S1 and S2). For further analysis, the metabolites with variable importance in the projection (VIP) value > 1.0 in PLS1 or PLS2 were selected and *p*-value < 0.05 were selected. Significance (*p*-value < 0.05) between selected metabolites, their relative contents, and the values from the antioxidant activity and physicochemical properties assays was tested by analysis of variance (ANOVA) and Duncan’s multiple range tests using PASW Statistics 18.0 software (SPSS Inc., Chicago, IL, USA). Heat map and correlation map were visualized using MEV software. 

### 3.6. Determination of Antioxidant Activity (by ABTS), Total Polyphenol Content (TPC), and Total Flavonoid Content (TFC)

ABTS, TPC, and TFC were conducted using assay described by Jung et al. with slight modifications [59]. For the ABTS assay, a dark-blue-colored stock solution was prepared by dissolving 7 mM ABTS in a 2.45 mM potassium persulfate solution, incubating the solution in a water bath at 60 °C for 20 min, and storing this solution for 12 h at room temperature. The solution was then diluted to reach an absorbance 0.7 ± 0.03 at 750 nm using a microplate reader (Spectra MAX190, Molecular Devices, San José, CA, USA). Then, each extracted sample (10 µL) was added to the diluted ABTS solution (190 µL) in a 96-well plate. After 7 min in dark, absorbance was measured at 750 nm using a microplate reader. Trolox was used as a standard, and the result was showed as the Trolox equivalent antioxidant capacity (TEAC), with the standard solution concentration curve ranging from 0.015 mM to 1000 mM. 

For the TPC assay, the Folin–Ciocalteu colorimetric method was used. Briefly, 0.2 N Folin–Ciocalteu’s phenol reagent (100 µL) was added to 20 µL of each sample in a 96-well plate, followed by incubation at room temperature for 6 min in the dark. Next, 80 µL of 7.5% sodium carbonate solution was added to the mixture, reacted for 60 min at room temperature, and measured at 750 nm. The result was presented as the gallic acid equivalent (GAE) concentration (ppm) per milligram of FSP in a standard solution concentration curve ranged between 31.25 ppm and 500 ppm. 

The TFC was determined by measuring the absorbance of the reacted samples placed in 96-well plates. Briefly, 180 µL 90% diethylene glycol (C_4_H_10_(OH)_3_), 20 µL of 1 N sodium hydroxide (NaOH), and 20 µL of each extracted sample were mixed and reacted at room temperature for 60 min in the dark. Next, the absorbance was evaluated at 405 nm using a microplate reader. The result was expressed as the naringin equivalent concentration (NE). The standard solution concentration curve ranged between 15.625 ppm and 200 ppm. All experiments were performed in triplicate.

### 3.7. Evaluation of Physicochemical Characteristics

Physicochemical characteristics were evaluated as described by Lee et al. with some modifications [60]. An FSP sample (3 g) was mixed with 30 mL of distilled water, homogenized on a Twist Shaker (Biofree, Seoul, Korea) for 60 min, and centrifuged at 8000× *g* for 5 min at 4 °C. Then, the supernatants were collected and filtered using 0.22-μm PTFE filters before assays for reducing sugar contents, salinity, pH, titratable acidity, and amino-type nitrogen.

Reducing sugar content (300 µL per each) was determined using portable digital refractometer for measurement of sugar (HI 96811, Hanna Instruments, Inc., Padova, Italy). Salinity (300 µL per each) were determined using a portable digital refractometer for measurement of sodium chloride (HI 96821, Hanna Instruments, Inc., Padova, Italy).

Each sample pH was determined by using a pH meter (Thermo Fisher Scientific, Waltham, MA, USA). Titratable acidity and amino-type nitrogen contents were estimated using the formol titration method, as described previously by Lee et al. [60]. Total acidity was considered by titrating the FSP sample extract with 0.1 N sodium hydroxide (NaOH) (pH 8.4). The consumed volume of sodium hydroxide solution (*V*_a_) was calculated into percent acetic acid using the following formula:

Titratable acidity (%) = [(0.006 × *V*_a_ × *D* × *F*)/*S*] × 100 (1)

The coefficients of the formula are represented that 0.006 is conversion factor for acetic acid, *V*_a_ is the consumption amount for sodium hydroxide (mL), *D* is the dilution rate (1), *F* is factor of the 0.1 N sodium hydroxide solution (1.002), and S is amount of sample (3g).

After supplementing 20 mL of formaldehyde solution (37%), amino-type nitrogen contents were evaluated by re-titrating to pH 8.4, using 0.1 N sodium hydroxide solutions (*V*_a_). The milligram percentage of amino-type nitrogen contents was calculated using the following formula:

Amino-type nitrogen (% mg) = [(*V*_a_ × 1.4 × *D* × *F*)/*S*] × 100. (2)

The coefficients of the formula are represented that *V*_a_ is the consumed volume of NaOH solution (mL) and, 1.4 is the nitrogen equivalent amount of the 0.1 N NaOH solution (1 mL). 

## 4. Conclusions

In this study, we comprehensively investigated the metabolic repertoire, antioxidant activities, and physicochemical properties of six FSPs. We conjecture that the primary metabolite abundance in the six FSPs was largely affected by their respective substrate ingredients. Further, the secondary metabolite contents were mainly influenced by the substrate ingredients, microbial assortments, and the fermentation period, with latter being the most important factor. As metabolites contribute to savor, nutrition, and bioactivity in foods, the study may help toward fermented food quality improvement. Further, the study may suggest the important biomarkers for quality control in commercial FSPs.

## Figures and Tables

**Table 1:** Information for the six kinds of FSPs ^a^.

Sample No.	Product	General Main Ingredient			General Fermentation Period	Producer
		Soybean	Cereal	Chili pepper		
1	Natto (NT)	99.99%	-	-	18 h	Shikaya Co., LTD
2						Suzusei Shokuhin Co., LTD
3						Azuma Shokuhin Co., LTD
4						Marumiya Co., LTD
5						Takanofoods Co., LTD
6	Cheonggukjang (CG)	95%–99%	-	-	2 days	YoungPyung
7						Seoilfarm
8						PulDangGol
9						JukHyangKong
10						ChungHo food
11	Doenjang (DJ)	80%–95%	-	-	1~3 years	Sambou
12						MooSooChon
13						Greensoy
14						Koreamac
15						SunJae food
16	Miso (MS)	60%	25%^c^	-	2~12 months	Marusanai CO., LTD
17						Miyasaka Jozo CO., LTD
18						Hanamaruki Foods Inc.
19						Masuyamiso CO., LTD
20						Marukome CO., LTD
21	Doubanjiang (DB)	20%^b^	5%^d^	40%–50%	over 1 year	Lee Kum Kee CO., LTD
22						Shandong Shinho Food Industries CO., LTD
23						Haha Jiang Yuan CO., LTD
24						Sichuan Dandan Pixian Bean Paste Group CO., LTD
25						Kikkoman Corporation
26	Tianmianjiang (TM)	0%–30%	20%–40% ^d^	-	over 1 year	Hsien Erh Mei Foods Industry CO., LTD
27						Weihai Sihai Brewed CO., LTD
28						Shandong Shinho Food Industries CO., LTD
29						Tian Jin Limin Condiment CO., LTD
30						Youki Food CO., LTD

^a^ Fermented soybean products (FSPs), ^b^ mixed with soybean and broad bean, ^c^ rice, ^d^ wheat.

**Table 2:** Comparison of bioactivity phenotypes and physicochemical characteristics in the six FSPs.

	NT	CG	DJ	MS	DB	TM
TPC ^f^	68 ± 5.88^a^	63 ± 11^a,b^	54 ± 4.44^a,b^	57 ± 8.30^a,b^	50 ± 16^b^	54 ± 10^a,b^
TFC ^g^	32 ± 5.00^a^	32 ± 12^a^	16 ± 2.42^b^	10 ± 4.43^b^	18 ± 7.24^b^	21 ± 12^a,b^
Reducing sugar content ^h^	1.94 ± 0.11^d^	1.54 ± 0.29^d^	3.22 ± 0.23^b^	3.92 ± 0.46^a^	2.58 ± 0.78^c^	4.44 ± 0.27^a^
Salinity ^i^	1.94 ± 0.11^d^	1.54 ± 0.23^d^	3.22 ± 0.18^b^	3.36 ± 0.39^a^	2.24 ± 0.65^c^	3.78 ± 0.23^a^
pH ^j^	7.16 ± 0.28^a^	6.66 ± 0.14^b^	5.72 ± 0.15^c^	5.42 ± 0.05^d^	4.51 ± 0.29^e^	4.56 ± 0.25^e^
Titratable acidity ^k^	6.07 ± 1.17^b^	4.49 ± 1.73^b^	10.19 ± 1.12^a^	9.56 ± 1.85^a^	6.07 ± 2.15^b^	11.31 ± 2.31^a^
Amino type nitrogen content ^l^	2976 ± 705^b^	1336 ± 433^c^	6161 ± 848^a^	2634 ± 679^b^	1790 ± 1519^b,c^	2368 ± 346^b,c^

f, Expressed as naringin equivalent concentration (NE) (ppm): g, Expressed as gallic acid equivalent concentration (GE) (ppm): h, Expressed as Brix%: i, Expressed as mg%: j, Expressed as pH: k, Expressed as g/100 mL: l, Expressed as % acid. All the values were averaged for FSP types. Different letters (a–e) in the table indicate significant difference determined by ANOVA, followed by Duncan’s multiple-range test (*p*-value < 0.05).

## Supplementary Materials

Supplementary materials can be accessed at: https://www.mdpi.com/2218-1989/9/9/183/s1, Figure S1: Heat map representations for significantly discriminant primary metabolites and secondary metabolites, showing correlations with soybean content, antioxidant activity (ABTS), total phenolic content (TPC), total flavonoid content (TFC), reduced sugar content, salinity, pH, titratable acidity, and amino-type nitrogen content. Table S1: List of significantly different metabolites according to six FSPs based on GC-TOF-MS data, Table S2: List of significantly different metabolites according to six FSPs based on LC-MS data, Table S3: List of the sample product and producer name.
