# Supplementary material for: Comparative Evaluation of Six Traditional Fermented Soybean Products in East Asia: A Metabolomics Approach
Source: Metabolites. 2019 Sep 13;9(9):183. doi: 10.3390/metabo9090183 (PMC6780719; doi:10.3390/metabo9090183)
Supplement: Supplementary file 1 [file metabolites-09-00183-s001.pdf]

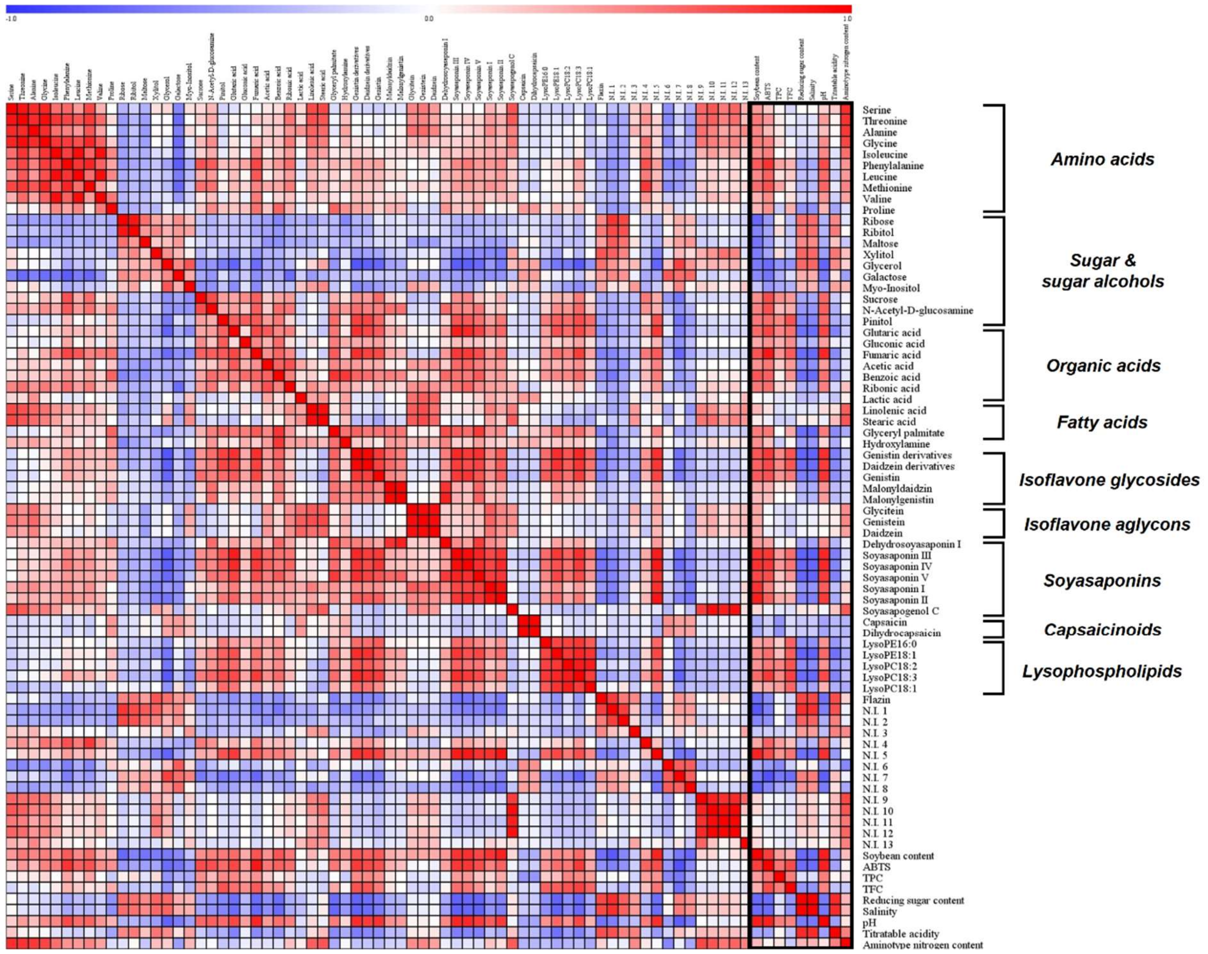

Figure S1. Heat map representations for significantly discriminant primary metabolites and secondary metabolites, showing correlations with soybean content, antioxidant activity (ABTS), total phenolic content (TPC), total flavonoid content (TFC), reduced sugar content, salinity, pH, titratable acidity, and amino-type nitrogen content. N.I., non-identification.

Table S1 List of significantly different metabolites according to the six FSPs based on GC-TOF-MS data.

No.	Tentative Metabolites [a]	RT [b] (min)	MS Fragment pattern (*m*/*z*)	Identified ion [c] (*m*/*z*)	TMS [d]	ID [e]
			Amino acids			
1	Serine	8.03	73, 204, 218, 100, 147, 75	204	3	MS
2	Threonine	8.28	73, 117, 219, 218, 57, 147	219	3	MS
3	Alanine	5.40	147, 73, 116, 204, 148, 59	116	2	STD
4	Glycine	7.52	174, 73, 147, 86, 175, 59	174	3	MS
5	Isoleucine	6.20	86, 73, 75, 69, 87, 74	86	1	STD
6	Phenylalanine	10.30	73, 218, 192, 100, 147, 75	218	2	STD
7	Leucine	5.97	86, 75, 73, 74, 87, 146	170	1	STD
8	Methionine	9.42	73, 128, 147, 176, 74, 157	128	2	MS
9	Valine	5.22	72, 75, 73, 55, 74, 146	72	1	STD
10	Proline	7.45	142, 73, 107, 256, 140, 77	256	2	STD
			Sugar & sugar derivatives			
11	Ribose	14.77	73, 147, 98, 103, 217, 160	160	4	STD
12	Ribitol	15.96	73, 103, 147, 156, 217, 446	446	5	STD
13	Maltose	17.20	73, 147, 204, 361, 217, 103	361	8	STD
14	Xylitol	11.04	73, 217, 147, 103, 129, 117	217	5	STD
15	Glycerol	7.21	73, 147, 117, 103, 205, 133	205	3	STD
16	Galactose	12.33	73, 147, 103, 117, 205, 129	118	5	STD
17	Myo-Inositol	13.59	73, 147, 217, 191, 305, 129	305	6	STD
18	Sucrose	16.55	73, 236, 230, 147, 245, 192	236	4	MS
19	N-Acetyl-D-glucosamine	13.53	73, 147, 129, 202, 87, 205	202	4	MS
20	Pinitol	11.88	73, 147, 217, 133, 260, 191	260	5	MS
			Organic acids			
21	Glutaric acid	9.84	129, 73, 147, 247, 75, 157	129	3	MS
22	Gluconic acid	13.04	73, 147, 103, 333, 74, 217	333	5	MS
23	Fumaric acid	7.84	147, 245, 133, 246, 174, 149	245	2	STD
24	Acetic acid	5.10	147, 73, 66, 177, 148, 75	177	2	MS
25	Benzoic acid	6.89	179, 105, 135, 77, 51, 180	51	1	MS
26	Ribonic acid	11.50	73, 147, 292, 103, 217, 75	292	5	MS
27	Lactic acid	4.95	73, 117, 147, 66, 59, 133	88	2	STD
			Fatty acids			
28	Linolenic acid	14.19	75, 73, 79, 55, 129. 67	116	1	MS
29	Stearic acid	14.29	117, 75, 73, 129, 132, 145	117	1	MS
30	Glyceryl Palmitate	16.18	73, 147, 57, 55, 371, 203	371	2	MS
			Etc.			
31	Hydroxylamine	5.56	73, 133, 146, 119, 147, 86	146	3	MS

a Tentatively identified metabolites were selected as differential variables based on p-value < 0.05 and VIP 1, 2 > 1.0, VIP 1 and VIP 2, value of variable importance in projection from PLS1 and PLS2 of PLS-DA model; b RT Retention time; c *m*/*z*, the selected ion for identification; d TMS, trimethylsilyl; e ID, identification. STD, standard compounds. MS, the mass spectra of NIST and in-house libraries.

Table S2 List of significantly different metabolites according to the six FSPs based on LC-M

No.	Tentative Identificatioins [a]	UHPLC-LTQ-ESI-IT-MS/MS					UPLC-L	
		RT [b] (min)	Measured mass(m/z)		MS^n fragments(m/z) [c]	λmax(nm)	Measured mass(m/z)	El Co
			[M+H]$^+$	[M-H]$^-$				
					Isoflavones			
32	Genistein derivatives	8.03	533	531	533>271>243,215,153>197,187	264	533.12575	C2
33	Daidzein derivatives	7.33	517	515	517>255>227,199,137>181,171	268(sh) [f]	517.13140	C2
34	Genistin	6.78	433	477 [e]	433>271>243,215,153	260,319(sh)	433.11069	C2
35	Malonyldaidzin	7.06	503	547 [e]	503>255>227,199,137>181	266,366(sh)	503.11585	C2
36	Malonylgenistin	7.83	519	517	519>271>243,215,153>197,187	264,366(sh)	519.11036	C2
37	Glycitein	8.50	285	283	285>270>242>214	214,270(sh)	285.07405	C1
38	Genistein	9.43	271	269	271>253,243,215,153	218,258(sh)	271.05825	C1
39	Daidzein	8.29	255	253	255>227,199,137>181>153	210,259	255.06354	C1
					Soyasaponins			
40	Dehydrosoyasaponin I	11.26	941	985 [e]	941>795,597,439	222	941.50457	C4
41	Soyasaponin III	11.07	797	795	797>599,441>581,423>297,255	221	797.46322	C4

Table S2 Cont.

No.	Tentative Identificatioins [a]	UHPLC-LTQ-ESI-IT-MS/MS					UPLC-LTQ-Orbitrap-MS/MS				I.D. [d]
		RT [b] (min)	Measured mass(*m/z*) [M+H]+	Measured mass(*m/z*) [M-H]-	MS^n fragments(*m/z*) [c]	λmax(nm)	Measured mass(m/z)	Elemental Composition	RDB	Delta ppm	
					Soyasaponins						
42	Soyasaponin IV	11.20	767	812 [e]	767>599,441>423	222	767.45250	C41H66O13	8.5	-6.669	LIB
43	Soyasaponin V	10.64	959	957	959>599>441>581,423>405, 281	219	959.51409	C48H78O19	9.5	-7.208	Ref [3]
44	Soyasaponin I	10.75	943	987	943>797,599,441>581,423>3 35,267	221	943.52028	C48H78O18	9.5	-6.160	LIB
45	Soyasaponin II	11.02	913	911	913>895,781,617,423	221	913.50943	C47H76O17	9.5	-6.674	LIB
46	Soyasapogenol C	15.40	441	-	441>423>405,365,283,203	224	441.36967	C30H48O2	6.5	-6.881	-
					Capsaicinoids						
47	Capsaicin	11.91	306	-	306>170>137	222	306.20444	C18H27NO3	5.5	-6.304	Ref [5]
48	Dihydrocapsaicin	12.53	308	-	308>184,137>109	222	308.22012	C18H29NO3	4.5	-6.165	Ref [5]
					Lysophospholipids						
49	LysoPE16:0	13.48	454	452	454>436,313>393	223	454.28997	C21H44NO7P	0.5	6.230	Ref [4]
50	LysoPE18:1	13.84	480	478	480>462,339>419,265	223	480.30555	C23H46NO7P	1.5	-6.071	-
51	LysoPC18:2	13.11	520	564 [e]	521>502,183>443>263,163	223	520.33628	C26H50NO7P	2.5	-6.699	Ref [4]
52	LysoPC18:3	12.46	518	562 [e]	518>500>441>305,181	222	518.32070	C26H48NO7P	3.5	-6.590	Ref [4]
53	LysoPC18:1	13.92	522	566 [e]	522>504,184>445>309,265,1 63	224	522.35213	C26H52NO7P	1.5	-6.291	Ref [2]
					Etc.						
54	Flazin	8.68	309	307	309>291>281>263,222	216,271(sh)	309.08515	C17H12N2O4	12.5	-5.932	-

Table S2 Cont.

No.	Tentative Identificatioins [a]	UHPLC-LTQ-ESI-IT-MS/MS					UPLC-LTQ-Orbitrap-MS/MS				I.D. [d]
		RT [b] (min)	Measured mass(*m/z*)		MS^n fragments(*m/z*) [c]	λmax(nm)	Measured mass(m/z)	Elemental Composition	RDB	Delta ppm	
			[M+H]$^+$	[M-H]$^-$							
					Non-Identifications						
55	N.I. 1	1.15	276	274	276>258>210>164	276(sh)	276.14244	C12H21NO6	2.5	-6.243	-
56	N.I. 2	1.42	425	423	425>407>389>341	271	425.20046	C20H28N2O8	7.5	-5.250	-
57	N.I. 3	6.08	245	-	245>217,119>172,70	268(sh)	245.18464	C12H24N2O3	1.5	-5.421	-
58	N.I. 4	9.27	733	731	733>602>489>461,376	211,260	733.48137	C41H66O10N	9.0	7.391	-
59	N.I. 5	9.69	927	971 [e]	927>811,613,455>595,437	219	927.49048	C49H70N2O15	15.5	6.021	-
60	N.I. 6	11.00	274	-	274>256>102	221	274.27192	C16H35NO2	-0.5	-7.787	-
61	N.I. 7	11.86	376	420 [e]	376>302,293>275	222	376.25703	C21H33N3O3	6.5	-6.481	-
62	N.I. 8	16.52	256	-	256>186,115>88	225	256.26149	C16H33NO	0.5	-7.809	-
63	N.I. 9	16.82	428	426	428>382,166>120	224	428.31289	C27H41NO3	7.5	-7.076	-
64	N.I. 10	16.90	394	392	394>376,348,131>292	225	394.32863	C24H43NO3	3.5	-7.458	-
65	N.I. 11	17.49	370	368	370>352,324,132	225	370.32932	C22H43NO3	1.5	-6.078	-
66	N.I. 12	17.70	396	394	396>378,350,132>333,263,212	225	396.34478	C24H45NO3	2.5	-6.158	-
67	N.I. 13	17.88	384	-	384>367,302,245,204,122	226	384.32332	C26H41NO	6.5	-7.211	-

a Tentatively identified metabolites were selected as differential variables based on *p*-value < 0.05 and VIP 1, 2 > 1.0; VIP 1 and VIP 2, value of variable importance in projection from PLS1 and PLS2 of PLS-DA model; b RT, Retention time; c MS^n fragment patterns detected in positive mode; d I.D., identification; LIB, In-house library; Ref., References; e Adduct ion is formic acid, [M+COOH]$^-$; f sh, Shoulder; N.I., non-identification.

References

1. Kudou, S.; Tsuizaki, I.; Uchida, T.; Okubo, K. Purification and some properties of soybean saponin hydrolase from *Aspergillus oryzae* KO-2. *Agric. Biol. Chem*. **1991**, *55(1)*, 31-36.
2. Seo, H.S.; Lee, S.; Singh, D.; Shin, H.W.; Cho, S.A.; Lee, C.H. Untargeted metabolite profiling for *koji*-fermentative bioprocess unravels the effects of varying substrate types and microbial inocula. *Food chem.* **2018**, *266*, 161-169.
3. Lee, S.Y.; Lee, S.; Lee, S.; Oh, J.Y.; Jeon, E.J.; Ryu, H.S.; Lee, C.H. Primary and secondary metabolite profiling of *doenjang*, a fermented soybean paste during industrial processing. *Food chem.* **2014**, *165*, 157-166
4. Lee, G.M.; Suh, D.H; Jung, E.S.; Lee, C.H. Metabolomics provides quality characterization of commercial *gochujang* (fermented pepper paste). *Molecules*. **2016**, *21*(7), 921.
5. Jang, Y.K.; Shin, G.R.; Jung, E.S.; Lee, S.; Lee, S.; Singh, D.; Jang, E.S.; Shin, D.J; Kim, H; Shin, H.W.; Moon, B. S.; Lee, C.H. Process specific differential metabolomes for industrial *gochujang* types (pepper paste) manufactured using white rice, brown rice, and wheat. *Food chem.* **2017**, *234*, 416-424.
6. Baiocchi, C.; Medana, C.; Giancotti, V.; Aigotti, R.; Dal Bello, F.; Massolino, C.; Gastaldi, D.; Grandi, M. Qualitative characterization of Desmodium adscendens constituents by high-performance liquid chromatography-diode array ultraviolet-electrospray ionization multistage mass spectrometry. *Eur J Mass Spectrom.* **2013**, *19*(1), 1-15.

Table S3. List of the sample product and producer name

Sample No.	Product	Product name	Producer
1	Natto (NT)	Shikaya Ganko Oyaji Kotsubu Natto	Shikaya Co., LTD
2		Suzusei Natto	Suzusei Shokuhin Co., LTD
3		Azuma Natto Oraganic	Azuma Shokuhin Co., LTD
4		Power kid Natto	Marumiya Co., LTD
5		Okame Natto	Takanofoods Co., LTD
6	Cheonggukjang (CG)	bamboo salt Cheonggukjang	YoungPyung
7		SeoBunRae Cheonggukjang	Seoilfarm
8		PulDangGol Cheonggukjang	PulDangGol
9		DanYang Cheonggukjang	JukHyangKong
10		JeonYoungJu Cheonggukjang	ChungHo food
11	Doenjang (DJ)	bamboo salt Doenjang (Sujak)	Sambou
12		MooSooChon Doenjang	MooSooChon
13		Jeju blue soybean Doenjang	Greensoy
14		Mac Doenjang	Koreamac
15		Tradiotional Doenjnag	SunJae food
16	Miso (MS)	Awase Miso	Marusanai CO., LTD
17		Shinsyu-ichi Miso	Miyasaka Jozo CO., LTD
18		Okasan Miso (Awase)	Hanamaruki Foods Inc.
19		Aawase Miso	Masuyamiso CO., LTD
20		Siro Miso	Marukome CO., LTD
21	Doubanjiang (DB)	Chili bean sauce (Toban Djan)	Lee Kum Kee CO., LTD
22		Cong Ban Lv Soybean Paste	Shandong Shinho Food Industries CO., LTD
23		Har Har Hot bean sauce	Haha Jiang Yuan CO., LTD
24		Szechuan Doubanjiang	Sichuan Dandan Pixian Bean Paste Group CO., LTD
25		Doubanjiang	Kikkoman Corporation
26	Tianmianjiang (TM)	Tianmianjiang	Hsien Erh Mei Foods Industry CO., LTD
27		Hogumi Tianmianjiang	Weihai Sihai Brewed CO., LTD
28		Cong Ban Lv Tianmianjiang	Shandong Shinho Food Industries CO., LTD
29		Tianmianjiang	Tian Jin Limin Condiment CO., LTD
30		Tianmianjiang	Youki Food CO., LTD
